# Supplementary material for: High-throughput methods leveraging robotics and computer vision for the development of therapeutic phage cocktails
Source: Nat Commun. 2026 Jan 30;17:2192. doi: 10.1038/s41467-026-68684-x (PMC12960834; doi:10.1038/s41467-026-68684-x)
Supplement: Supplementary file 1 — Supplementary Information [file 41467_2026_68684_MOESM1_ESM.pdf]

High-throughput methods leveraging robotics and computer vision for the development and assessment of bacteriophage therapeutic cocktails - Supplementary Information

Taylor J.R. Penke[1*], Aeron Tynes Hammack[1,2*], Lana McMillan[1], Nick Healy[1,3], Morgan K.Y. Wall[1,2], Pearl Wilcock[1], Ethan Baker[1], Naomi Chavez[1], Iain Wright[1], Hannah H. Tuson[1], Ashley Trama[1], Cameron J. Prybol[1], Eyra Dordi[1], Ava Ghobadian[1], David G. Ousterout[1], Nicholas R. Conley[1], Paul Garofolo[1]

[1*]Research and Development, Locus Biosciences, 523 Davis Drive, Morrisville, 27560, North Carolina, United States.
[2*]Molecular Foundry, Lawrence Berkeley National Laboratory, 1 Cyclotron Road, Berkeley, 94720, California, United States.
[3]Automation Research, Hamilton Company, 4970 Energy Way, Reno, 89502, Nevada, United States.

*Corresponding author(s). E-mail(s): taylor.penke@locus-bio.com; athammack@lbl.gov;
Contributing authors: lana.mcmillan@locus-bio.com; TBD; mkwall@lbl.gov; pearlwilcock@gmail.com; ethan.baker@locus-bio.com; naomi.chavez@locus-bio.com; imwright@ncsu.edu; hannah.tuson@locus-bio.com; ashley.trama@locus-bio.com; cameron.prybol@locus-bio.com; erdordi@gmail.com; ava.ghobadian@locus-bio.com; dave.ousterout@gmail.com; nick.conley@locus-bio.com; paul.garofolo@locus-bio.com;

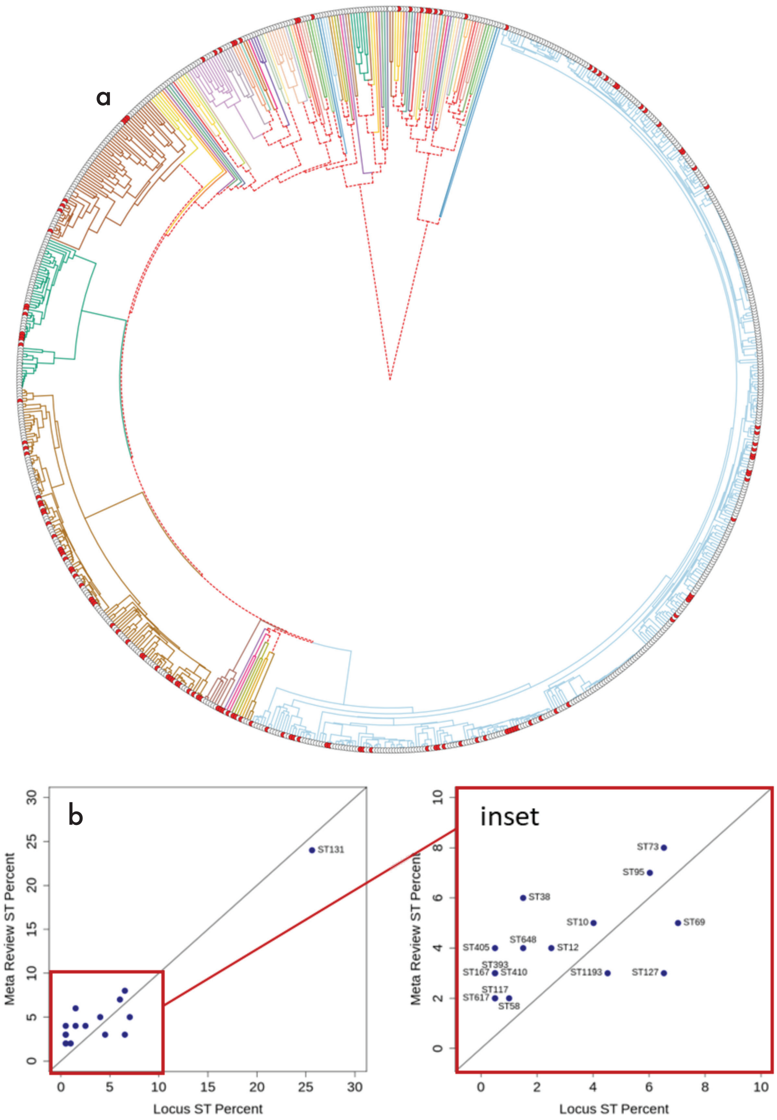

Supplementary Figure 1 Meta Analysis of Strains. a. 10,000 *E.coli* genomes were downloaded from the BV-BRC (formerly PATRIC) database [1] and filtered for genome completeness. Genomes of internally banked strains were collected and clustered alongside public genomes using aKronyMer to generate a genome distance matrix [2]. Approximately 1000 genomes were randomly selected from the above and represented as a dendrogram using `ggdendro`. Nodes were colored white for public genomes and red for internal genomes. Most clusters were represented by at least one internal genome. **b.** Common sequencing types (ST) observed in extraintestinal pathogenic bacteria were collected from a meta review of ST studies. Scatterplot shows the observed percentage of ST on UTI Clinical Panel compared to that observed in the metareview [3].

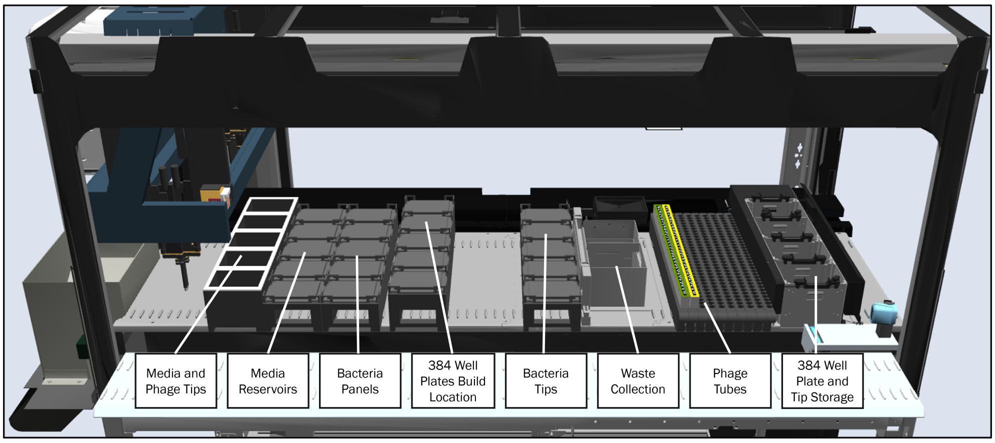

Supplementary Figure 2 Schematic of automated optical density time course phage efficacy assay deck layout. This figure shows the organization of automated carriers on the Hamilton VANTAGE used to run the optical density time course based phage efficacy assay. White boxes indicate the labware used on each carrier. Instrument movements were optimized to minimize traversals of used tips over biospecimens. In the assay 384-well plates are moved from the storage elevator to the build location and delidded. Media is added to each well using the 96-channel pipettor, and subsequently, phages are added to the plate using the individual 8 channels. During the phage addition, the robotic arm moves tips from the storage elevator to the bacteria tips carrier. After phage addition is completed, bacteria is added to the 384-well plates using the 96-channel pipettor. 384-well plates are then relidded and moved to shaking incubators using the robotic arm. Image reproduced with permission from Hamilton Company. © 2025 Hamilton Company.

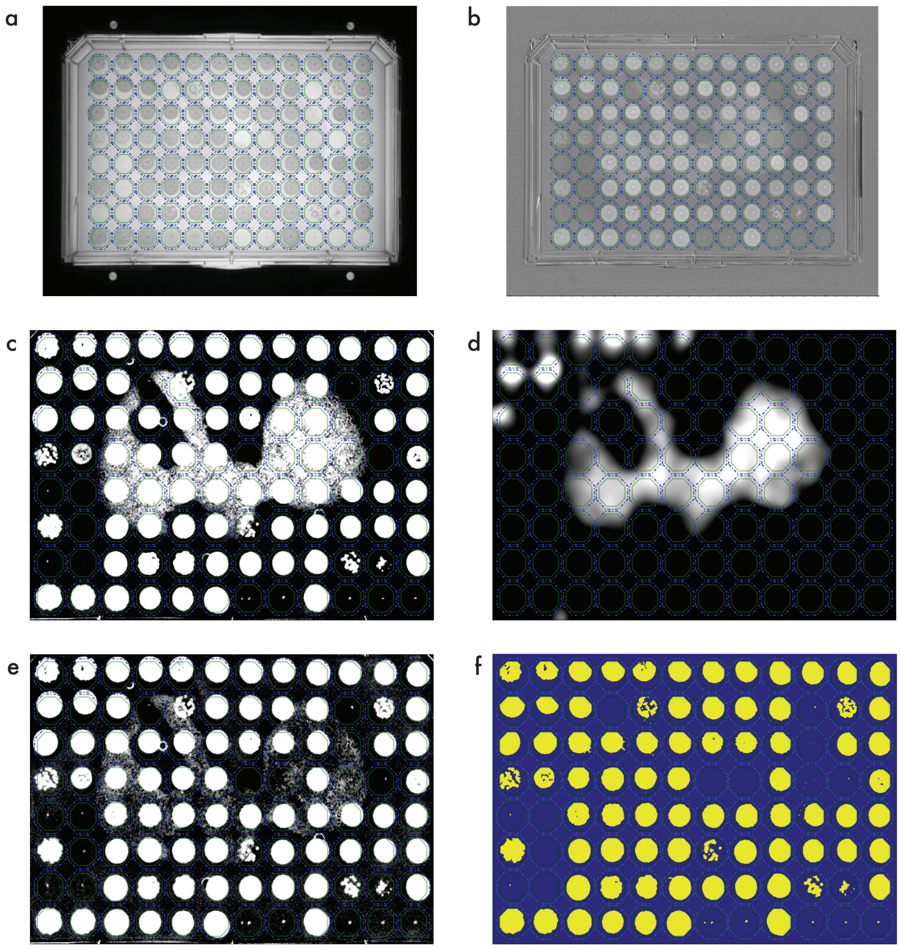

Supplementary Figure 3 Background Subtraction Example. a. Raw unprocessed image with regions of interest (ROIs) overlaid. **b.** The same image as in panel **a**, but after the averaged blank image has been subtracted and the image inverted to account for the fact that it is a CFU sample. **c.** The same image after cropping and median filtering with a 5×5 pixel kernel. **d.** The background model formed from the triangular meshed sampling of image pixels outside of the ROIs. **e.** The resulting fully background-subtracted image to be subsequently enumerated. **f.** The thresholded regions of the image that are expected to contain bacterial colonies to be enumerated.

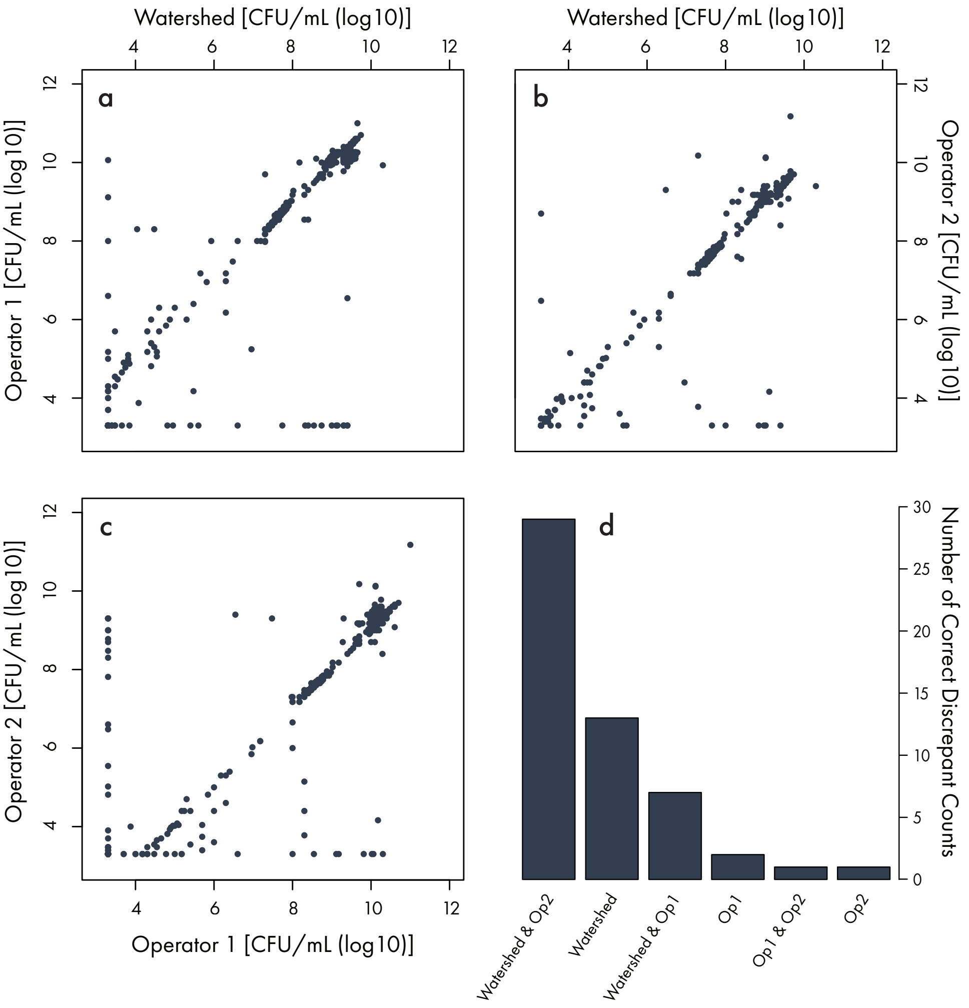

Supplementary Figure 4 Comparison of automated to manual colony counting. 768 titer series were counted by the automated enumeration pipeline and manually by two independent operators (Op). Scatterplots show the $\log_{10}$ Colony Forming Units (CFU)/mL values for the automated pipelines compared to each analyst (top) and the two analysts compared to one another (bottom left). All counts that varied by greater than 1.5 log were assessed by a third manual counter to tie break discrepancies. For each of the 53 discrepant counts an assessment was performed to determine which counting method was correct. Correct counts were defined as those that aligned with the tie breaker and where 3 counting methods were aligned within 1 log. **d.** Bar-chart showing the total number of times that each counting method, or combination of methods, was correct by this metric. "$A\,\&\,B$" (e.g. "Watershed & Op2") indicates both A and B were correct, where A and B are different counting methods. The total counts of all bars sum to the total 53 discrepant counts.

References

[1] Gillespie, J. J. *et al.* PATRIC: the comprehensive bacterial bioinformatics resource with a focus on human pathogenic species. *Infect. Immun.* **79**, 4286–4298 (2011).

[2] Al-Ghalith, G. & Knights, D. akronymer enables database-free metagenome comparison (2018).

[3] Manges, A. R. *et al.* Global extraintestinal pathogenic escherichia coli (ExPEC) lineages. *Clin. Microbiol. Rev.* **32** (2019).
