# Supplementary material for: High-throughput methods leveraging robotics and computer vision for the development of therapeutic phage cocktails
Source: Nat Commun. 2026 Jan 30;17:2192. doi: 10.1038/s41467-026-68684-x (PMC12960834; doi:10.1038/s41467-026-68684-x)
Supplement: Supplementary file 2 — Reporting Summary [file 41467_2026_68684_MOESM2_ESM.pdf]

nature portfolio

Corresponding author(s): Taylor Penke, Aeron Hammack

Last updated by author(s): 13-Nov-2025

Reporting Summary

Nature Portfolio wishes to improve the reproducibility of the work that we publish. This form provides structure for consistency and transparency in reporting. For further information on Nature Portfolio policies, see our Editorial Policies and the Editorial Policy Checklist.

Statistics

For all statistical analyses, confirm that the following items are present in the figure legend, table legend, main text, or Methods section.

n/a	Confirmed	
☐	☒	The exact sample size (*n*) for each experimental group/condition, given as a discrete number and unit of measurement
☐	☒	A statement on whether measurements were taken from distinct samples or whether the same sample was measured repeatedly
☐	☒	The statistical test(s) used AND whether they are one- or two-sided<br>*Only common tests should be described solely by name; describe more complex techniques in the Methods section.*
☒	☐	A description of all covariates tested
☐	☒	A description of any assumptions or corrections, such as tests of normality and adjustment for multiple comparisons
☐	☒	A full description of the statistical parameters including central tendency (e.g. means) or other basic estimates (e.g. regression coefficient) AND variation (e.g. standard deviation) or associated estimates of uncertainty (e.g. confidence intervals)
☐	☒	For null hypothesis testing, the test statistic (e.g. *F*, *t*, *r*) with confidence intervals, effect sizes, degrees of freedom and *P* value noted<br>*Give P values as exact values whenever suitable.*
☒	☐	For Bayesian analysis, information on the choice of priors and Markov chain Monte Carlo settings
☒	☐	For hierarchical and complex designs, identification of the appropriate level for tests and full reporting of outcomes
☒	☐	Estimates of effect sizes (e.g. Cohen's *d*, Pearson's *r*), indicating how they were calculated

Our web collection on statistics for biologists contains articles on many of the points above.

Software and code

Policy information about availability of computer code

Data collection	The automation code developed for this publication is included at the following DOI: https://doi.org/10.5281/zenodo.17594564
Data analysis	The automation code developed for this publication is included at the following DOI: https://doi.org/10.5281/zenodo.17594564

For manuscripts utilizing custom algorithms or software that are central to the research but not yet described in published literature, software must be made available to editors and reviewers. We strongly encourage code deposition in a community repository (e.g. GitHub). See the Nature Portfolio guidelines for submitting code & software for further information.

Data

Policy information about availability of data

All manuscripts must include a data availability statement. This statement should provide the following information, where applicable:

- Accession codes, unique identifiers, or web links for publicly available datasets
- A description of any restrictions on data availability
- For clinical datasets or third party data, please ensure that the statement adheres to our policy

All data used for plotting is included in a supplementary table and at DOI https://doi.org/10.5281/zenodo.17594564. The remaining data developed for this clinical asset is proprietary, however, we can share with reviewers under embargo against publication or sharing.

Research involving human participants, their data, or biological material

Policy information about studies with human participants or human data. See also policy information about sex, gender (identity/presentation), and sexual orientation and race, ethnicity and racism.

Reporting on sex and gender	No reporting was performed on sex or gender as part of this manuscript. Clinical isolates were obtained from females as part of a sex-based trial inclusion criteria.
Reporting on race, ethnicity, or other socially relevant groupings	No reporting on race, ethnicity, or other socially relevant groupings occurred in this manuscript. Self-reported race/ethnicity are reported in the manuscript describing the results of the clinical trial from which these isolates were collected (10.1016/S1473-3099(24)00424-9)
Population characteristics	Bacterial isolates reported on in this manuscript were not analyzed based on the population characteristics of the patients originally hosting them.
Recruitment	Patient recruitment is based on enrollment criteria detailed as part of NCT05488340.
Ethics oversight	Bacterial isolates were collected as part of NCT05488340. The ethical oversight for this trial was provided as follows. The study protocol and informed consent form were approved by the central Advarra Institutional Review Board, which is compliant with all applicable laws and regulations and ethical principles for human subject research including The Belmont Report, Nuremberg Code, and Declaration of Helsinki. All eligible patients were informed about the study procedures and risks and provided written informed consent before enrollment. The informed consent forms included the following language regarding the collection of clinical isolates: Individual bacterial or phage isolates recovered from you may be retained for perpetuity and may lead to new tests, drugs, devices, or other products or services with commercial value

Note that full information on the approval of the study protocol must also be provided in the manuscript.

Field-specific reporting

Please select the one below that is the best fit for your research. If you are not sure, read the appropriate sections before making your selection.

- [x] Life sciences
- [ ] Behavioural & social sciences
- [ ] Ecological, evolutionary & environmental sciences

For a reference copy of the document with all sections, see nature.com/documents/nr-reporting-summary-flat.pdf

Life sciences study design

All studies must disclose on these points even when the disclosure is negative.

Sample size	Data was collected from all clinical isolates collected as part of NCT05488340 detailed in DOI: 10.1016/S1473-3099(24)00424-9
Data exclusions	Non- E. coli isolates were excluded from data collection.
Replication	Reproducibility of the experimental findings was verified through a strategic emphasis on biological breadth and scale, rather than conventional replication within a single experimental setup.
Randomization	No randomization was performed. All bacterial test entities were exposed to all treatment conditions.
Blinding	The individual analyzing the results of robotic and manual counts was blinded.

Reporting for specific materials, systems and methods

We require information from authors about some types of materials, experimental systems and methods used in many studies. Here, indicate whether each material, system or method listed is relevant to your study. If you are not sure if a list item applies to your research, read the appropriate section before selecting a response.

Materials & experimental systems

n/a	Involved in the study	
x		Antibodies
x		Eukaryotic cell lines
x		Palaeontology and archaeology
x		Animals and other organisms
x		Clinical data
x		Dual use research of concern
x		Plants

Methods

n/a	Involved in the study	
x		ChIP-seq
x		Flow cytometry
x		MRI-based neuroimaging

Plants

Seed stocks	N/A
Novel plant genotypes	N/A
Authentication	N/A
